# A promoter variant in ZNF804A decreasing its expression increases the risk of autism spectrum disorder in the Han Chinese population

**DOI:** 10.1038/s41398-019-0369-x

**Published:** 2019-01-22

**Authors:** Linna Zhang, Yue Qin, Xiaohong Gong, Rui Peng, Chunquan Cai, Yufang Zheng, Yasong Du, Hongyan Wang

**Affiliations:** 1 Department of Child & Adolescent Psychiatry, Shanghai Mental Health Center, Shanghai Jiao Tong University School of Medicine, Shanghai, 200030 China; 2 Obstetrics and Gynecology Hospital, State Key Laboratory of Genetic Engineering at School of Life Sciences, Institute of Reproduction and Development, Fudan University, Shanghai, 200011 China; 3 Ministry of Education (MOE) Key Laboratory of Contemporary Anthropology, School of Life Sciences, Fudan University, Shanghai, 200438 China; 4 Department of Neurosurgery, Tianjin Children’s Hospital, Tianjin, 300134 China; 5 Key Laboratory of Reproduction Regulation of NPFPC, Collaborative Innovation Center of Genetics and Development, Fudan University, Shanghai, 200032 China; 6 Children’s Hospital of Fudan University, Shanghai, 201102 China

## Abstract

Synaptic pathology may be one of the cellular substrates underlying autism spectrum disorder (ASD). ZNF804A is a transcription factor that can affect or regulate the expression of many candidate genes involved in ASD. It also localizes at synapses and regulates neuronal and synaptic morphology. So far, few reports have addressed possible associations between *ZNF804A* polymorphisms and ASD. This study aimed to investigate whether *ZNF804A* genetic variants contribute to ASD susceptibility and its possible pathological role in the disorder. We analyzed the relationship of two polymorphisms (rs10497655 and rs34714481) in *ZNF804A* promoter region with ASD in 854 cases versus 926 controls. The functional analyses of rs10497655 were then performed using real-time quantitative polymerase chain reaction, electrophoretic mobility shift assays, chromatin immunoprecipitation and dual-luciferase assays. The variant rs10497655 was significantly associated with ASD (*P* = 0.007851), which had a significant effect on *ZNF804A* expression, with the T risk allele homozygotes related with reduced *ZNF804A* expression in human fetal brains. HSF2 acted as a suppressor by down-regulating *ZNF804A* expression and had a stronger binding affinity for the T allele of rs10497655 than for the C allele. This was the first experiment to elucidate the process in which a disease-associated SNP affects the level of *ZNF804A* expression by binding with the upstream regulation factor HSF2. This result indicates that the rs10497655 allelic expression difference of *ZNF804A* during the critical period of brain development may have an effect on postnatal phenotypes of ASD. It reveals new roles of *ZNF804A* polymorphisms in the pathogenesis of psychiatric disorders.

## Introduction

Autism spectrum disorder (ASD) is characterized by early-onset deficiencies in social interactions as well as restricted and repetitive behaviors, interests or activities^1^. According to a report from the United States in 2012, the prevalence of this pervasive developmental disorder has increased to 1 in 68 children^2^. The Centers for Disease Control and Prevention (CDC of the USA) reported that ASD occurs in all racial, ethnic, and socioeconomic groups but is approximately 4.5 times more common among boys than among girls. ASD is a neurodevelopmental disorder, with an estimated heritability of 0.7–0.9 based on large-scale studies^3,4^. The genetic etiology of ASD has been proven to be complex and heterogeneous, with over 800 genes implicated in this disease (https://www.sfari.org/resource/sfari-gene/), as shown by various studies including genome-wide association studies (GWAS)^5,6^, whole exome sequencing (WES)^7,8^, association studies or functional analyses of candidate genes for single nucleotide polymorphisms (SNP)^9–11^ or rare mutations^12,13^ and the detection of copy number variations (CNV)^14,15^.

Increasing evidence shows that synaptic pathology may be one of the cellular substrates underlying ASD^16^. Recently, it has been found that ZNF804A/Zfp804A localizes at synapses and regulates neuronal and synaptic morphology^17^. *ZNF804A* is a gene that encodes a transcription factor which contains zinc finger and nucleic acid binding domains. It can affect or regulate the expression of many candidate genes involved in ASD, such as *PDE4B* and *DRD2*^18–21^. Moreover, the expression of *ZNF804A* in the brain has been revealed to be significantly decreased in individuals with ASD than controls^22^. *ZNF804A* was also found to be a risk gene for ASD by CNV analyses^23–25^. Therefore, the identification and evaluation of correlation between *ZNF804A* and ASD is of great value. So far, few reports have addressed possible associations between *ZNF804A* polymorphisms and ASD, except that Anitha and colleagues reported the intronic SNP rs7603001 of *ZNF804A* was related to verbal skills in individuals with ASD^22^. It is necessary to investigate more potential ASD-associated variations in the *ZNF804A* gene.

In order to determine whether *ZNF804A* genetic variants in the promoter contribute to ASD susceptibility and its possible pathological role in the disorder, two common polymorphisms with minor allele frequency (MAF) > 10% in the promoter region were tested for association with ASD in 854 Chinese ASD cases and 926 controls. Our results demonstrated that rs10497655 was associated with ASD susceptibility and the T allele significantly increases the risk of ASD. Furthermore, functional assays were performed to explore its role of rs10497655 in the pathogenesis of this disease.

## Materials and Methods

### Ethics statement

Written informed consent was obtained from the controls and the guardians or the parents of the children with ASD. This study was approved by the Ethics Committee of the Shanghai Mental Health Center, Shanghai Jiao Tong University School of Medicine and the Ethics Committee of the School of Life Sciences, Fudan University. Ethical approval for the collection and the distribution of the brain samples for research was obtained from Tianjin Children’s Hospital.

### Study subjects

Samples of 854 patients with ASD (751 males and 103 females) and 926 controls (817 males and 109 females) were recruited separately from Department of Child and Adolescent Psychiatry, Shanghai Mental Health Center, Shanghai Jiao Tong University School of Medicine (Shanghai, China) and Fudan University (Shanghai, China) between February 2010 and January 2014. All of the subjects collected were ethnic Han Chinese. All of the controls recruited from Fudan University were matched to the patients according to geography and gender. The average ages (years) of the ASD patients and the controls were 5.91 ± 0.15 and 18.58 ± 0.01 (mean ± SE), respectively. Of the patients, 670 individuals recruited from 2010 to 2013 were diagnosed with ASD according to the criteria of Diagnostic and Statistical Manual of Mental Disorders, fourth Edition (DSM-4) of the American Psychiatric Association (APA) and 184 individuals recruited from 2013 to 2014 were diagnosed with ASD according to DSM-5^1^. Based on the DSM-4 or DSM-5 scores on overall levels of social ability (scores of 0–4 for DSM-4 and scores of 0–3 for DSM-5), repetitive behavior (scores of 0–4 for DSM-4 and DSM-5) or language (scores of 0–4 for DSM-4 only), individuals with ASD were divided into mild deficiency (scores of 0, 1, or 2) subgroups and severe deficiency (scores of 3 or 4) subgroups.

### Genotyping of two common variants in the *ZNF804A* gene

Between 1 and 3 mL of venous blood was collected from each individual in this study. Genomic DNA was isolated using the Mammalian Genomic DNA Extraction Kit (Lifefeng, Shanghai, China). Two promoter SNPs with MAF > 10% (rs10497655 and rs34714481) were genotyped by sequencing in 854 ASD cases and 926 controls. All primer pairs for sequencing were designed using the online software PRIMER 3 (http://bioinfo.ut.ee/primer3–0.4.0/) and are shown in Table S1. For the sequencing reaction, PCR products were pre-treated with a mixture of 10 units of Exo I and 1 unit of FastAP (Thermo Scientific, MA, USA). BigDye Terminator v3.1 was used for the direct sequencing reaction according to the manufacturer’s instructions (Applied Biosystems, CA, USA). HiDi formamide (Applied Biosystems, CA, USA) was used in the subsequent denaturation, and finally, the samples were submitted for sequencing using an ABI 3730xl sequencer (Applied Biosystems, CA, USA). The results were analyzed with Genalys software (version 2.8.3).

### Real-time quantitative polymerase chain reaction (RT-qPCR) in brain samples

Eighteen human fetal brain tissues from terminations of pregnancy were provided by Tianjin Children’s Hospital. All were second-trimester fetal brain samples that had no obvious defects. Genomic DNA and total RNA were extracted simultaneously from all samples using the TRNzol reagent (TIANGEN BIOTECH, Beijing, China). Each DNA sample was genotyped for the SNP rs10497655. The primer pairs are listed in Table S1.

RNA samples were treated with gDNase for 3 min to remove genomic contamination and then first-stranded cDNA was synthesized using the FastQuant RT kit (with gDNase) (TIANGEN BIOTECH, Beijing, China). *ZNF804A* mRNA levels were measured by quantitative real-time PCR using the StepOne system (Applied Biosystems, CA, USA). The standard reaction mixture contained 10 μM of each primer, the SuperReal PreMix Plus (SYBR Green) kit (TIANGEN BIOTECH, Beijing, China) and 2 μl of cDNA (1:10 dilution). *hGAPDH* was used as an internal reference gene. Relative *ZNF804A* mRNA levels were measured independently with two pairs of primers targeting different regions. All of the samples were finished for RT-qPCR with oligonucleotides hZNF804A-F1/R1. Each reaction was performed in triplicate at least four times. The primers are listed in Table S1.

### Electrophoretic mobility shift assays (EMSA)

Nuclear proteins were extracted from HEK-293T cells using NE-PER nuclear and cytoplasmic extraction reagents (Pierce, IL, USA). Duplex oligonucleotide probes representing the -1052 C or T alleles of rs10497655 (sequences listed in Table S1) were labeled with biotin. The assays were performed using a LightShift Chemiluminescent EMSA kit (Thermo Scientific, MA, USA) according to the manufacturer’s protocols. Briefly, 50 fmols of biotin-labeled duplex oligonucleotides bearing either the -1052 C or the T allele of rs10497655 were incubated with 5 μg of nuclear extracts for 20 min in 10 × binding buffer supplemented with 1 µg/µl poly (dI·dC), 50% glycerol and 1% NP-40. Unlabeled probes at 5- or 50-fold molar excesses, as indicated, were added to the reaction for competition. The reaction mixture was then electrophoresed on a native 6% polyacrylamide gel and transferred to a positive nylon membrane. The detection of biotin-labeled DNA was performed using stabilized streptavidin-horseradish peroxidase conjugate nd the membrane was exposed to X-ray film. Each experiment was repeated three times.

### Chromatin immunoprecipitation (ChIP) assays

Human neuroblastoma IMR-32 cells were grown in DMEM supplemented with 10% FBS (Gibco, CA, USA) and 1‱ Plasmocin (InvivoGen, CA, USA). ChIP assays were performed using nuclear extracts obtained from IMR-32 cells heterozygous for C > T at rs10497655 in the *ZNF804A* promoter. For each reaction, approximately 2 × 10^6^ IMR-32 cells were cross-linked with 1% formaldehyde at room temperature for 10 min, followed by termination with glycine added to the sample for another 5 min. The chromatin was then sonicated into fragments with a length of 200 to 750 bp using Bioruptor (Diogenode, Liege, Belgium). For immunoprecipitation, the sheared chromatin was incubated with anti-HSF2 (sc-13056×) antibody (Santa Cruz Biotechnology, CA, USA) or non-specific rabbit IgG (Invitrogen, CA, USA) for 4 h at 4 °C. Finally, the purified DNA fragments were identified by PCR (primers see Table S1) that caught a 254-bp-specific product that covered the variant C > T. To further investigate the difference in the binding affinity between the C and T alleles of the SNP rs10497655, the ratio of the two alleles was quantified using SNaPshot from the ChIP input and the purified DNA precipitated with anti-HSF2. The SNaPshot products were detected on an ABI 3730xl automated sequencer and analyzed with Peak scanner software (version 1.0). All values were normalized to the input levels. Three independent assays were performed.

### Plasmid construction

To construct the *ZNF804A* promoter luciferase reporter plasmid, the 1148-bp fragment from −1715 to −568 of human *ZNF804A* was amplified from genomic DNA using PCR, which contains the T allele of the -1052 C > T SNP (rs10497655). The PCR products were subcloned into the *Mlu*I and *Xho*I restriction sites of the pGL3-Basic vector (Promega, WI, USA), in which the firefly luciferase gene was used as a reporter. The corresponding plasmid containing the C allele was generated by site-directed mutagenesis using the MutanBEST kit (Takara, Dalian, China). Human *HSF2* cDNA was donated by Professor Han (Xiamen University, Xiamen, China). The *HSF2* CDS fragments were amplified with KOD PLUS (Toyobo, Shanghai, China) and cloned into the *EcoR*V and *Kpn*I restriction sites of the pcDNA3.1(-) vector (Invitrogen, CA, USA).We verified all of the plasmids by bidirectional sequencing. The primers used are shown in Table S1.

### Cell culture, transfection and luciferase reporter assays

Human embryonic kidney HEK-293T cells and human neuroblastoma SK-N-AS cells were grown in DMEM supplemented with 10% FBS (Corning, NY, USA) and 1‱ Plasmocin (InvivoGen, CA, USA). For the *ZNF804A* promoter activity study, 80% confluent HEK-293T and SK-N-AS cells in the 24-well plate were co-transfected with 500 ng of ZNF804A promoter reporter plasmid carrying the C or the T allele of rs10497655 and 10 ng of the pRL-TK plasmid (Promega, WI, USA), in which the *Renilla* luciferase gene was used as an internal control. Furthermore, the cells were additionally co-transfected with 200 ng of the pcDNA3.1(-)-HSF2 expression plasmid or equivalent amounts of the empty pcDNA3.1(-) vector. Lipofectamine 2000 or 3000 (Invitrogen, CA, USA) transfection reagent was used for the transfections, according to the manufacturer’s instructions. After 24 h of culture, the transfected cells were lysed and 20 µl of the supernatant was assayed for luciferase activity using the Dual-Luciferase Reporter Assay System (Promega, WI, USA). The relative reporter activity was obtained by normalization of the firefly activity to *Renilla* activity. Each assay was performed in triplicate, and each experiment was performed at least three times.

### Bioinformatics analysis

Potential transcription factors (TFs) that bind the sites around the specific target SNP rs10497655 were computationally predicted using the online software TFSEARCH (http://www.cbrc.jp/research/db/TFSEARCH.html).

### Statistical analysis

The statistical analysis was conducted with R software (Version 3.1.3) and GraphPad Prism software (Version 5.04, GraphPad Software Inc., USA), which was also used for graphics. Differences in allelic or genotypic frequencies between the ASD cases and the controls were compared by the χ^2^ test, which was also used to assess the Hardy–Weinberg equilibrium (HWE) for the controls. To evaluate the associations between genotypes and ASD risk, odds ratios (ORs) and 95% confidence intervals (CIs) were calculated using a logistic regression analysis. The online software SNPStats (http://bioinfo.iconcologia.net/snpstats/start.htm) was used to analyze the association between different haplotypes and ASD risk. In RT-qPCR, fold changes were calculated using the 2^−ΔΔCt^ method and presented as normalized fold expression. The Mann–Whitney *U* test was used to evaluate the differences in *ZNF804A* relative expression levels between the two defined groups. Student’s *t*-test was used to compare the values of SNaPshot assays. A one-way ANOVA test was applied for luciferase assays and co-transfection. A repeated-measures ANOVA test was also used in the assays co-transfected with the HSF2 plasmid or the pcDNA3.1(−) empty vector. In graphics, the quantitative variables are given as the mean ± SE. All statistical tests were two-tailed, with a significance level of 0.05.

## Results

### The *ZNF804A* promoter variant rs10497655 C > T significantly increases the risk of ASD

We searched for common genetic variants with MAF > 10% and identified two SNPs (rs10497655 and rs34714481) within the promoter region of *ZNF804A* in the case-control cohort. We analyzed the relationship of rs10497655 or rs34714481 with the risk of ASD in 854 cases versus 926 controls. The genotype distribution of two SNPs were in Hardy–Weinberg equilibrium in controls. It was showed that strong association with ASD was observed for the SNP rs10497655 (*P* = 0.007851, Table 1). The minor T allele was significantly related to a higher risk for ASD (OR = 1.20, 95% CI = 1.05–1.37). On the other hand, no association was detected between SNP rs34714481 and ASD (*P* = 0.2381). The genotype frequencies of rs10497655 showed significant differences between ASD cases and controls in codominant, recessive and overdominant models (*P* = 2.12 × 10^−5^, *P* = 2.23 × 10^−5^, *P* = 1.35 × 10^−4^, respectively, Table 2). Compared with the CC and CT genotypes, the TT genotype of rs10497655 was significantly more common in the ASD cases than in the controls (OR = 1.58, 95% CI = 1.28–1.95). Individuals with ASD were divided into mild deficiency and severe deficiency categories based on social ability, language and repetitive behaviors, separately. A stronger association of the SNP rs10497855 was observed in ASD individuals with severe deficiency on language (*P* = 0.0006, OR = 1.37, 95% CI = 1.14–1.63, Table 3), whereas no association was observed in other categories considering multiple test corrections (Table 3). Compared with the CC-CT genotypes, the TT genotype was significantly more common in ASD individuals with severe deficiency on language than in the controls (*P* = 1.00 × 10^–4^, OR = 1.90, 95% CI = 1.45–2.50). No gender differences were observed for allele frequencies of rs10497655 between controls and cases when males or females were considered separately (data was not shown).

**Table 1:** Allele frequencies of two SNPs in the ASD patients and controls

SNP ID	Allele	Control, *n*%	Case, *n*%	*P* value^a^	OR (95% CI)^b^	MAF^c^			
						Control	Case	CHB^d^	CHS^d^
rs10497655	C	963(52.0%)	811(47.5%)	0.007851	1.20(1.05–1.37)	0.48	0.53	0.495	0.476
	T	889(48.0%)	897(52.5%)						
rs34714481	A	1234(66.6%)	1105(64.7%)	0.2381	1.08(0.95–1.24)	0.33	0.35	0.286	0.319
	G	618(33.4%)	603(35.3%)						

*OR* odds ratio, *CI* confidence interval

^a^Differences between cases and controls under allele frequencies were compared using the chi-square test with 1 degree of freedom (d*f*)

^b^ORs and 95% CIs were calculated by the logistic regression analysis

^c^MAF indicates minor allele frequency

^d^They represent allelic frequencies in the Chinese Han populations based on data from the 1000 Genomes Project

**Table 2:** Associations between rs10497655 and ASD in the case-control study

SNP ID	Genetic model	Pattern	Control, *n*%	Case, *n*%	*P* value^a^	HWE *P* val^b^	OR (95% CI)^c^
rs10497655	Codominant	C/C	250(27.0%)	231(27.0%)	2.12 × 10^–5^	0.9997	Reference
		C/T	463(50.0%)	349(40.9%)			0.82(0.65–1.02)
		T/T	213(23.0%)	274(32.1%)			1.39(1.08–1.79)
	Dominant	C/C	250(27.0%)	231(27.0%)	1		Reference
		C/T-TT	676(73.0%)	623(73.0%)			1.00(0.81–1.23)
	Recessive	C/C–C/T	713(77.0%)	580(67.9%)	2.23 × 10^–5^		Reference
		T/T	213(23.0%)	274(32.1%)			1.58(1.28–1.95)
	Overdominant	C/C–T/T	463(50.0%)	505(59.1%)	1.35 × 10^-4^		Reference
		C/T	463(50.0%)	349(40.9%)			0.69(0.57–0.83)

^a^Differences between cases and controls under different genetic models were compared using the chi-square test with 2 degrees of freedom (df) or 1 degree of freedom (d*f*)

^b^HWE *P* val indicates *P* value for the Hardy–Weinberg equilibrium test in the control subjects

^c^OR indicates the odds ratio; CI, confidence interval. ORs and 95% CIs were calculated by the logistic regression analysis

**Table 3:** Allele frequencies of rs10497655 in the controls and ASD subgroups

Allele	Control, n%	MDS^a^	SDS^b^	MDR^c^	SDR^d^	MDL^e^	SDL^f^
C	963(52.0%)	263(48.2%)	488(48.1%)	533(48.7%)	218(46.8%)	286(50.7%)	291(44.2%)
T	889(48.0%)	283(51.8%)	526(51.9%)	561(51.3%)	248(53.2%)	278(49.3%)	367(55.8%)
*P* value^#^		0.1157	0.0475	0.0856	0.0441	0.5918	0.0006
OR (95% CI)*		1.17 (0.96–1.41)	1.17 (1.00–1.36)	1.14 (0.98–1.32)	1.23 (1.01–1.51)	1.05 (0.87–1.27)	1.37 (1.14–1.63)

*OR* odds ratio, *CI* confidence interval

^#^Differences between cases and controls under allele frequencies were compared using the chi-square test with 1 degree of freedom (d*f*)

^*^ORs and 95% CIs were calculated by the logistic regression analysis

^a^Patients grouped into mild deficiency on social ability

^b^Patients grouped into severe deficiency on social ability

^c^Patients grouped into mild deficiency on repetitive behavior

^d^Patients grouped into severe deficiency on repetitive behavior

^e^Patients grouped into mild deficiency on language

^f^Patients grouped into severe deficiency on language

### The rs10497655 C > T variation decreases the transcriptional activity of *ZNF804A* in vivo

**Fig. 1:**
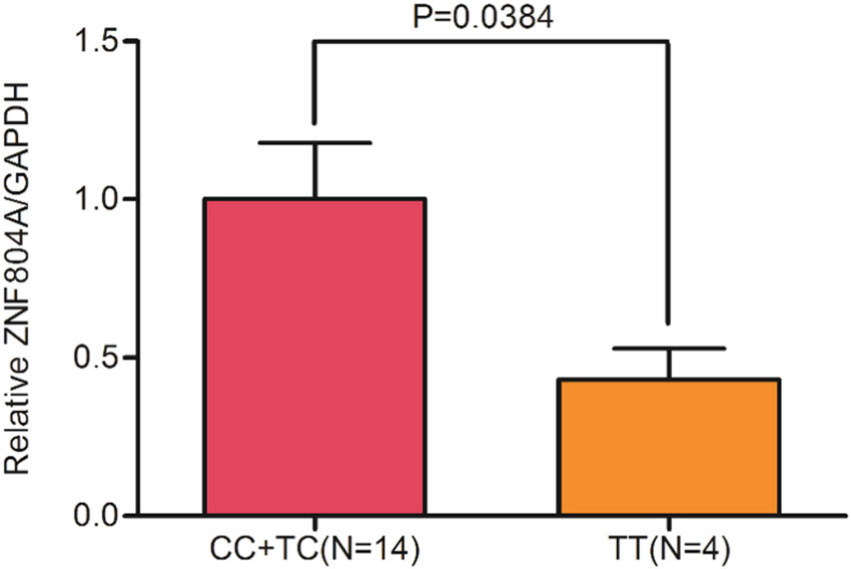
Quantitative real-time PCR analysis of the *ZNF804A* mRNA level in 18 brain tissue samples with different genotypes of rs10497655. All values were transformed with the level of human *GAPDH* mRNA as a reference before the analysis and then the data were normalized to the CC + TC group. The values for the groups are as follows: 1.0000 ± 0.1777 in group CC + TC and 0.4305 ± 0.0985 in group TT. The significance between the two groups was *P* = 0.0384, which was the result from the Mann–Whitney *U*-test conducted by GraphPad Prism. The data shown are described as the mean ± SE of at least four independent experiments

Since rs10497655 was located in the promoter region of the *ZNF804A* gene, different alleles may influence the transcription level of *ZNF804A* mRNA. To test this possibility, 18 post-mortem fetal brain samples from second-trimester abortions were tested for in vivo mRNA expression levels, with 8 CC genotypes, 6 CT genotypes and 4 TT genotypes. The promoter activity of the TT group was significantly lower than the CC + CT genotype group, which displayed approximately forty percent of the latter (Fig. 1). The relative *ZNF804A* mRNA levels of the brain samples were measured independently with two pairs of primers using the RT-qPCR method, and the two sets of data showed a similar trend (see Figure S1). Combined with the genetic data of rs10497655 in ASD, this result indicates that the effect of the risk T allele on ASD is mediated by reducing the expression of *ZNF804A*. This may be induced by the interaction of the rs10497655 C > T variation with certain inherent factors inside cells that greatly influence the transcription process of *ZNF804A*.

### The variant rs10497655 alters the binding capacity with nuclear proteins in the promoter region

**Fig. 2:**
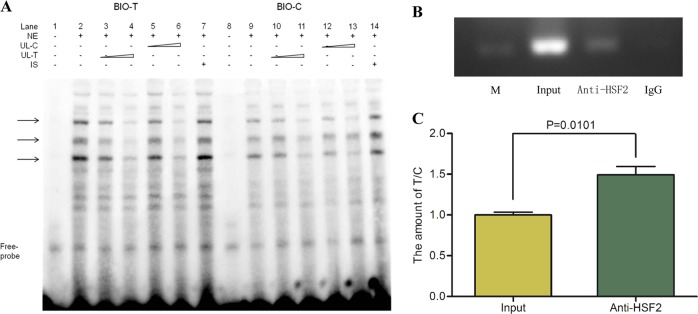
**a** The difference in the combining capacity with HEK-293T nuclear proteins in the T and C allele probes shown in EMSA. Lanes 1–7: biotin-labeled probe containing the T allele plus HEK-293T nuclear extract, except for lane 1; lanes 8–14: biotin-labeled probe containing the C allele and nuclear proteins, except for lane 8; NE represents nuclear proteins isolated from HEK-293T cells; UL-C/T represents unlabeled C/T probes; IS represents the unlabeled probe with an irrelevant sequence. From lane 3 to 4, 5 to 6, 10 to 11, and 12 to 13, 5- to 50-fold excesses of the corresponding unlabeled probes were used. **b** Chromatin immunoprecipitation (ChIP) assays in IMR-32 cells with the specific antibody to the predicted transcription factor HSF2. The in vivo binding of the HSF2 protein to the rs10497655 position was verified with polymerase chain reaction (PCR). M indicates the 250-bp band of DNA markers; the other three lanes are products from PCR with the ChIP input, DNA precipitated by anti-HSF2 and DNA by IgG as templates. **c** The amounts of the T/C alleles were quantified by SNaPshot from the ChIP input and products treated anti-HSF2. The ratio of the T allele to the C in the DNA immunoprecipitated (1.4926 ± 0.1016) was higher than that in the input DNA (1.0000 ± 0.0346), and the difference reached statistical significance. All values were normalized to the input levels. Significance between two groups was *P* = 0.0101 < 0.05, which was the result from unpaired *Student*’s t-test performed by GraphPad Prism. Data shown are mean ± SE from three independent assays

To verify the binding capacity of transcription factors to the C or T alleles of rs10497655, EMSA was carried out with nuclear extract prepared from HEK-293T cells. The EMSA analysis showed that the 26-bp core sequence containing the T allele of rs10497655 had a higher binding affinity than the C allele (Fig. 2a, lanes 2 and 9). The specificity of the proteins bound to the fragment covering rs10497655 was proven by competition tests in which 5-/50-fold excess of unlabeled corresponding probes were added (Fig. 2a, lanes 3–4 and 12–13) and un-competition tests in which an unspecific probe was added (Fig. 2a, lane 7 and 14). A cross-competition experiment further confirmed the observation of a higher binding affinity of the probe containing the T allele to certain proteins than the C allele (Fig. 2a, lanes 5–6 and lanes 10–11).

### Direct interactions and different Binding Affinities of Transcription factor HSF2 to the C/T alleles of rs10497655

The EMSA results revealed that certain TFs bind to the SNP rs10497655 and show different affinities for the T/C alleles. The computational analysis (TFSEARCH) predicted that the alteration from C to T at rs10497655 may influence the binding affinity of HSF2 (Figure S2). To prove this prediction, we carried out ChIP assays using IMR-32 cell lines with a heterozygous genotype at rs10497655. The results showed that the region covering the variant rs10497655 was occupied specifically by HSF2 (Fig. 2b). In the quantification by SNaPshot assays, the ratio of T/C alleles in the DNA immunoprecipitated by anti-HSF2 (1.4926 ± 0.1016) was significantly higher than in the input DNA (*P* = 0.0101), indicating that the T allele of rs10497655 had an approximately 1.5-fold higher affinity with HSF2, compared to the C allele (Fig. 2c).

### HSF2 functions as a repressor in the regulation of *ZNF804A* expression

**Fig. 3:**
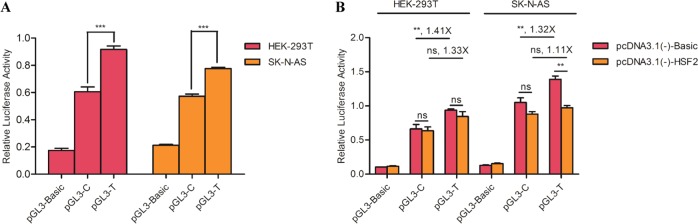
**a** Relative luciferase activity in the C or the T allele of the ZNF804A promoter. Luciferase expression was significantly higher in the T allele construct compared with the C allele construct in different cell lines. The values in the HEK-293T cells were as follows: pGL3-Basic = 0.1737 ± 0.0158, pGL3-C = 0.6062 ± 0.0351, pGL3-T = 0.9174 ± 0.0250. The values in the SK-N-AS cells were as follows: pGL3-Basic = 0.2117 ± 0.0074, pGL3-C = 0.5731 ± 0.0156, pGL3-T = 0.7765 ± 0.0095. B. HSF2 regulates ZNF804A expression as a repressor by interacting with rs10497655 loci. **a** Luciferase construct containing the C or the T allele was transfected together with pcDNA3.1(-)-Basic (control) or the pcDNA3.1(-)-HSF2 (expression vector) plasmid in different cell lines. In HEK-293T cells, the values of co-transfection with pcDNA3.1(-)-Basic were as follows: pGL3-Basic = 0.1032 ± 0.0017, pGL3-C = 0.6633 ± 0.0649, pGL3-T = 0.9368 ± 0.0187, values with pcDNA3.1(−)–HSF2: pGL3-Basic = 0.1149 ± 0.0080, pGL3-C = 0.6359 ± 0.0553, pGL3-T = 0.8463 ± 0.0690. In the SK-N-AS cells, the values of co-transfection with pcDNA3.1(−)-Basic were as follows: pGL3-Basic = 0.1270 ± 0.0072, pGL3-C = 1.0515 ± 0.0673, pGL3-T = 1.3898 ± 0.0482, values with pcDNA3.1(-)-HSF2: pGL3-Basic = 0.1529 ± 0.0087, pGL3-C = 0.8784 ± 0.0378, pGL3-T = 0.9729 ± 0.0337. Each value is shown as the mean ± SE. *** Denotes *P* < 0.001, **Denotes *P* < 0.01, ns represents no significance, which were the results from the one-way ANOVA or the repeated measures ANOVA test conducted by GraphPad Prism. The graphs above are representative of three independent experiments

Luciferase assays and co-transfection were performed to explore how HSF2 regulates the target promoter sequence. First, we tested the promoter activity of the region covering rs10497655 using luciferase assays, in which two cell lines, HEK-293T and SK-N-AS, were transfected with plasmids carrying the C or T allele of rs10497655 and an internal control plasmid carrying the *Renilla* luciferase gene. Both HEK-293T and SK-N-AS cell lines showed that the transcriptional activity of the fragment containing the T allele of rs10497655 was significantly higher than the C allele (Fig. 3a). Furthermore, the cells were additionally co-transfected with the pcDNA3.1(-)-HSF2 plasmid or the empty pcDNA3.1(-) vector. When co-transfected with the empty pcDNA3.1(-) vector, the T allele showed 1.41-fold transcriptional activity compared to the C allele., Although this divergence decreased, this result was no longer significant when the pcDNA3.1(-)-HSF2 plasmid existed (Fig. 3b). The co-transfection results in human neuroblastoma SK-N-AS cells showed that the overexpressed HSF2 decreased the ZNF804A transcription level, and its inhibition effect on the T allele was stronger than on the C allele. These data show that HSF2 acts as an inhibitor in *ZNF804A* transcription. In the RT-qPCR experiment, a lower *ZNF804A* mRNA level in individuals carrying the TT genotype can partly be explained by the stronger binding affinity and the inhibition effect of HSF2 on the T allele.

## Discussion

Compared with substantial reports of the relationship between *ZNF804A* and schizophrenia (SCZ)^26^, few studies have been reported on *ZNF804A* and ASD. *ZNF804A* CNVs have been found in individuals with ASD^6,22^, which indicates *ZNF804A* is involved in ASD. Anitha et al. reported that an intronic SNP rs7603001 was associated with autistic individuals who were verbally deficient. So far, most identified disease-associated SNPs are in introns, causing tremendous difficulties for function studies. To validate and further identify potential functional variations in *ZNF804A*, we performed an association study in ASD by testing two common SNPs in the promoter region. Our results showed that only one SNP rs10497655 was associated with ASD. What’s more, we also found the association of this variant with severe deficiency on language in ASD, consistent with the report of Anitha. The variant rs10497655 has been rarely reported, except in a schizophrenia research study in which no association was found between rs10497655 and SCZ^27^. The variant rs10497655 is located 1052 nucleic acids upstream of the transcription start site. A deep exploration was performed to test its potential effect on *ZNF804A* expression.

*ZNF804A* expression varies at different brain development times. Hill et al. discovered an allelic expression difference of *ZNF804A* in second-trimester, but not in first-trimester, human fetal brains, where the schizophrenia risk allele (T) of rs1344706 was associated with reduced *ZNF804A* expression^28^. The expression of *ZNF804A* was significantly reduced in specific brain regions in autistic individuals compared with controls^22^. In this study, the transcription levels of *ZNF804A* were quantified in second-trimester human fetal brains. Genotypes of the positive SNP rs10497655 had a significant effect on *ZNF804A* expression, with the TT homozygotes associated with reduced *ZNF804A* expression. This result indicates that the allelic expression difference of *ZNF804A* rs10497655 during the critical period of brain development may have an effect on the postnatal phenotype of ASD.

Although the differences in *ZNF804A* expression in the ASD cases and the controls and the allelic expression difference of *ZNF804A* rs1344706 were subsequently reported, the molecular mechanism was not clear. So far, only one report has found that a 5’UTR SNP rs359895 altered the binding affinity of transcription factor Sp1^27^. In our study, EMSA and ChIP assays showed that HSF2 had a stronger binding affinity for the T allele of rs10497655 than for the C allele. Luciferase reporter assays showed that HSF2 acted as a suppressor by down-regulating *ZNF804A* expression. This is the first report that HSF2 regulates *ZNF804A* expression by binding to a promoter SNP in *ZNF804A*.

HSF2 belongs to the family of heat shock transcription factors, which mediate cell reactions to stressful conditions. HSF1 is activated by environmental stresses such as heat shock and heavy metals and promotes the synthesis of heat shock proteins, thereby stabilizing intracellular homeostasis^29,30^. HSF2 regulates transcription by forming heterotrimers with HSF1 rather than directly responding to these environmental stresses^31^. The increased level of HSF2 is an activation signal for the following process. HSF2 is involved in corticogenesis and spermatogenesis^32^. Combined with our findings on the effect of HSF2 on *ZNF804A* expression, we speculate that in the critical stages of brain development, environmental stimuli exert influences on brain development via the *HSF2*-*ZNF804A* downstream gene pathway. Umeda-Yano et al., Girgenti et al. and Anitha et al. have confirmed that the upregulation or the knockout of *ZNF804A* can affect multiple downstream genes, including genes involved in neurodevelopment^18,22,33^. Intriguingly, *NLGN4* has been reported to be affected by ZNF804A regulation^17^. The role of *NLGN4* in ASD has been widely investigated using genetic, neurodevelopmental and biochemical methods^34^. The temporal and spatial expression characteristics of *ZNF804A* suggest that it is involved in brain development^35^. The regulation of *ZNF804A* in a series of genes including *NLGN4*, may be an important molecular mechanism for ASD. Our research links environmental stimuli and ASD through *HSF2-ZNF804A*. Obviously, this link is weak, but it provides an exciting new perspective for the understanding of the molecular mechanism of ASD. Experiments are warranted to investigate the alteration of the HSF2 level and its interaction with *ZNF804A* under various environmental conditions.

Accumulating evidence suggests that common genetic factors are shared among mental disorders, especially SCZ, bipolar disorder (BP) and ASD^36,37^. Abnormalities of brain morphology and function have been observed in SCZ, BP and ASD^38–50^, supporting aberrant neurodevelopment as the pathogenic mechanism of these disorders. The *ZNF804A* gene was first identified as a schizophrenia susceptibility gene at a genome-wide level^51^ and was subsequently reported to be associated with other psychiatric disorders such as bipolar disorder, autism spectrum disorder and heroin addiction^6,22,51–54^. As a transcription factor, *ZNF804A* may regulate neurodevelopment-related genes and therefore contributes to the pathogenesis of the disorders. Magnetic resonance imaging (MRI), cellular experiments and neurological data have shown that *ZNF804A* variants lead to brain structure and function changes^26,55^. The expression levels of genes that are involved in neurodevelopment and brain function such as *SNAP25*, *NLGN4*, *COMT* and *DRD2* are affected by *ZNF804A*^17,18,22^. In this study, we identified a promoter SNP, rs10497655, associated with ASD. Subsequently, *in vivo* and *in vitro* experiments showed that this variant alters the binding affinity for the environmental-sensitive regulation factor HSF2, and influences the allelic expression levels of *ZNF804A*. To our knowledge, this was the first experiment to elucidate the process in which a disease-associated SNP in the *ZNF804A* gene affects the level of *ZNF804A* expression by binding with the upstream regulation factor HSF2. Our findings provide new insights into the role of *ZNF804A* polymorphisms in the pathogenesis of psychiatric disorders.

Our findings support that *ZNF804A* is a risk gene for ASD in Han Chinese populations. The newly identified functional SNP (rs10497655) alters the binding affinity to for an the environmental- sensitive regulation factor HSF2, and influences allelic expression levels of *ZNF804A*. We provide the new evidence that *ZNF804A* genetic variants contribute to the susceptibility to ASD.

## Supplementary information

ZNF804A supplementary file
