# Supplementary material for: A promoter variant in ZNF804A decreasing its expression increases the risk of autism spectrum disorder in the Han Chinese population
Source: Transl Psychiatry. 2019 Jan 22;9:31. doi: 10.1038/s41398-019-0369-x (PMC6342935; doi:10.1038/s41398-019-0369-x)

**Supplementary file 1**

Table S1. DNA sequences of all used primer pairs

| Primer Name | Sequence (5’-3’) | Purpose |
| --- | --- | --- |
| ZNF804A-F1 | ACTCGGTTCCCCAGAAACAT | PCR/Sequence-PRO |
| ZNF804A-R1 | TATCCACTTGGGGGAGTCTG | PCR/Sequence-PRO |
| ZNF804A-F2 | TGCAAATTCTCGCTTTCTCC | PCR/Sequence-PRO |
| ZNF804A-R2 | TTGGTTTTCAAGGGTCCAAG | PCR/Sequence-PRO |
| ZNF804A-F3 | TTACACAAAGAGAGAATCAACTGG | PCR/Sequence-PRO |
| ZNF804A-R3 | CAAGTGGGAGACTCCTGGAA | PCR/Sequence-PRO |
| ZNF804A-PRO-CON-F | CCGACGCGTTTGACGTGTCCAGAGAATGC | Plasmid construct |
| ZNF804A-PRO-CON-R | CCGCTCGAGTTGCTAAAGCTGAGGGGAAA | Plasmid construct |
| ZNF804A-PRO-T to C-F | CAGAATGTCTGGAATCCGCTTTAACCAATTAAGC | Mutagenesis |
| ZNF804A-PRO-T to C-R | GCTTAATTGGTTAAAGCGGATTCCAGACATTCTG | Mutagenesis |
| HSF2-CDS-F | CCGGATATCGCCACCATGAAGCAGAGTTCGAACGT | Plasmid construct |
| HSF2-CDS-R | CGGGGTACCGAGCTATCTAAAAGTGGCATATC | Plasmid construct |
| EMSA-T-F | AATGTCTGGAATCTGCTTTAACCAAT | EMSA |
| EMSA-T-R | ATTGGTTAAAGCAAATTCCAGACATT | EMSA |
| EMSA-C-F | AATGTCTGGAATCCGCTTTAACCAAT | EMSA |
| EMSA-C-R | ATTGGTTAAAGCGGATTCCAGACATT | EMSA |
| IS PROBE-F | TGCAGTGAGCCAGTCTCCAGAGGACG | Irrelevant probe in EMSA |
| IS PROBE-R | CGTCCTCTGGAGACTGGCTCACTGCA | Irrelevant probe in EMSA |
| rs10497655-GT-F | GGTAACTTCCCCTTTCTACCATC | PCR/Genotype |
| rs10497655-GT-R | GGTTTTCAAGGGTCCAAGGC | PCR/Genotype |
| hGAPDH-F | TGCACCACCAACTGCTTAGC | RT-qPCR |
| hGAPDH-R | GGCATGGACTGTGGTCATGAG | RT-qPCR |
| hZNF804A-F1 | GCACCAGGAGTTTGACAATCACA | RT-qPCR |
| hZNF804A-R1 | CGAGCAAATTCCCTTTGTTTCAGTT | RT-qPCR |
| hZNF804A-F2 | GGACACTTTCGCAACATCAAGG | RT-qPCR |
| hZNF804A-R2 | CTTCCAGAGCTTTTGCTATGGTA | RT-qPCR |
| ChIP-PCR-F | ATGAGACACACAGAGTGGAATGT | ChIP |
| ChIP-PCR-R | TATACGATTCATCAGTCGGCACTT | ChIP |
| ChIP- SNaPshot | ttttttTGACATAGGGGCATCAGAATGTCTGGAATC | SNaPshot |

Figure S1. RT-qPCR assays of *ZNF804A* among most samples. Relative *ZNF804A* mRNA levels were measured with two kinds of primer pairs, hZNF804A-F1/R1 in A and hZNF804A-F2/R2 in B. The values in the assays with hZNF804A-F1/R1 are as follows: 1.0000±0.0899 in group CC+TC and 0.7015±0.1606 in group TT. The values in the assays with hZNF804A-F2/R2 are as follows: 1.0000±0.0819 in group CC+TC and 0.7325±0.1664 in group TT. The data shown are described as the mean ± SE.

A B

Figure S2. The computational analysis result of the prediction for transcription factors by the tool TFSEARCH. HSF2 was predicted as the possible transcription factor at the rs10497655 position by the online computational software TFSEARCH.


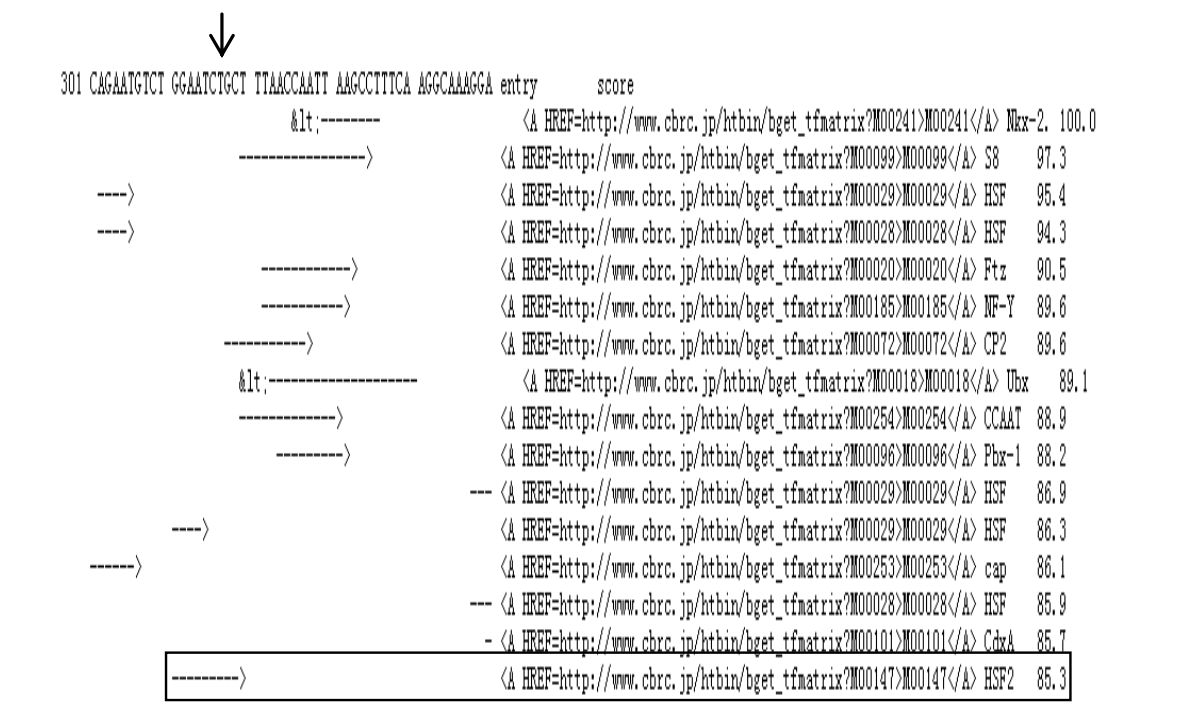

Supplement: Supplementary file 1 — ZNF804A supplementary file [file 41398_2019_369_MOESM1_ESM.docx]
